# Central blood pressure lowering effect of telmisartan‐rosuvastatin single‐pill combination in hypertensive patients combined with dyslipidemia: A pilot study

**DOI:** 10.1111/jch.14345

**Published:** 2021-08-12

**Authors:** JungMin Choi, Ki‐Chul Sung, Sang‐Hyun Ihm, Chang‐Hwan Yoon, Seung Woo Park, Sung‐Ha Park, Jang‐Young Kim, Sung‐Uk Kwon, Hae‐Young Lee

**Affiliations:** ^1^ Department of Internal Medicine Seoul National University Hospital Seoul Republic of Korea; ^2^ Division of Cardiology Department of Internal Medicine School of Medicine Kangbuk Samsung Hospital Sungkyunkwan University Seoul Republic of Korea; ^3^ Division of Cardiology Department of Internal Medicine College of Medicine The Catholic University of Korea Seoul Republic of Korea; ^4^ Cardiovascular Center & Department of Internal Medicine Seoul National University Bundang Hospital Seongnam Republic of Korea; ^5^ Division of Cardiology Department of Medicine Samsung Medical Center Heart Vascular Stroke Institute Sungkyunkwan University School of Medicine Seoul Republic of Korea; ^6^ Division of Cardiology Department of Internal Medicine Severance Cardiovascular Hospital Yonsei University College of Medicine Seoul Republic of Korea; ^7^ Department of Cardiology Wonju College of Medicine Yonsei University Wonju Republic of Korea; ^8^ Institute of Genomic Cohort Yonsei University Wonju Republic of Korea; ^9^ Department of Medicine Inje University Ilsan Paik Hospital Goyang Republic of Korea

**Keywords:** angiotensin II receptor blocker, dyslipidemia, hypertension, rosuvastatin, telmisartan

## Abstract

This multicenter, phase 4, Prospective Randomized Open, Blinded End‐point (PROBE) study aimed to evaluate safety and efficacy of telmisartan/rosuvastatin single‐pill combination (SPC) therapy on lowering central blood pressure (BP) compared with telmisartan monotherapy in hypertensive patients with dyslipidemia in Korea. Study was terminated earlier than planned due to COVID‐19 pandemic, thus should be considered as a pilot study. Among 125 patients who met the inclusion criteria of hypertension and dyslipidemia (defined as 10‐year Atherosclerotic Cardiovascular Disease risk score over 5%), 80 patients went through 4‐week single‐group run‐in period with telmisartan 40–80 mg, then randomized to telmisartan 80 mg + rosuvastatin (10 or 20 mg) SPC group or telmisartan 80 mg monotherapy group. The central/brachial BP, brachial‐ankle pulse wave velocity (baPWV), and augmentation index (AIx) were assessed at baseline and 16 weeks later. Mean brachial SBP changed from 135.80 ± 14.22 mmHg to 130.69 ± 13.23 mmHg in telmisartan/rosuvastatin group and from 134.37 ± 12.50 mmHg to 133.75 ± 12.30 mmHg in telmisartan monotherapy group without significant difference (between‐group difference *p *= .149). Mean central SBP were reduced significantly in the telmisartan/rosuvastatin group with change from 126.72 ± 14.44 mmHg to 121.56 ± 14.56 mmHg while telmisartan monotherapy group showed no significant change (between‐group difference *p *= .028). BaPWV changed from 1672.57 ± 371.72 m/s to 1591.75 ± 272.16 m/s in telmisartan/rosuvastatin group and from 1542.85 ± 263.70 m/s to 1586.12 ± 297.45 m/s in telmisartan group with no significance (between‐group difference *p *= .078). Change of AIx had no significant difference (between‐group difference *p *= .314). Both groups showed excellent compliance rate of 96.9 ± 4.5% with no significant difference in adverse rate. Telmisartan/rosuvastatin SPC therapy was more effective in lowering central BP compared with the telmisartan monotherapy. The results of this study showed benefit of additive statin therapy in hypertensive patients combined with dyslipidemia.

## 1 INTRODUCTION

Hypertension (HTN) is a known leading cause of death and disability over the world.^1^ Although lowering the blood pressure (BP) has been proven to reduce cardiovascular events,^2^ recent studies have suggested that the central BP might be more closely related to future cardiovascular events than brachial BP.^3^ Studies have shown stronger association of central BP with surrogate markers of cardiovascular diseases (eg, carotid intima media thickness,^4^ diastolic function,^5^ left ventricular mass^6^) and target organ damage (eg, renal failure,^7^ cognitive deficiency^8^) than brachial BP.

The central BP is a sum of incident wave from left ventricle and reflected wave from peripheral vessels at the ascending aorta. The measured contribution of reflected wave on the incident wave at the ascending aorta is defined as augmentation index (AIx).^9^ The AIx is affected by pulse wave velocity (PWV), which is defined by distance between two arterial sites divided by pulse transmit time.^10^ The AIx and PWV both have a predictive power on cardiovascular events.^11^, ^12^ Thus, current studies are also focusing on the predictive role of the AIx and PWV measurement as well as central BP.

Despite the rising importance of central BP, current antihypertensive medications are mainly focused on controlling brachial BP. However, it is well known that antihypertensive classes have different impact on central BP reduction. For example, beta‐blocker atenolol, was inferior in reducing central BP to brachial BP to other antihypertensive classes.^13^ And angiotensin‐converting enzyme inhibitors (ACEIs) and angiotensin receptor blockers (ARBs) have revealed more promising effect of lowering central BP than other classes of antihypertensives.^14^

Along with HTN, dyslipidemia is also known to increase the risk of cardiovascular events.^15^ The co‐existence of HTN and dyslipidemia is relatively abundant, with its prevalence of at least over 15%.^16^ The co‐existence leads to a higher risk of cardiovascular event than a simple sum of the two factors.^17^ Among the treatments of dyslipidemia, hydroxyl‐methyl‐glutaryl‐coenzyme A reductase inhibitors, statins, are the fundamental drug.^18^ Statins were reported to have modest BP lowering effect and is expected to have a beneficial role in arterial stiffening.^19^

Telmisartan is a highly selective blocker to angiotensin type‐1 receptor with a long elimination half‐life that effectively reduces BP for 24‐h dosage interval.^20^ Rosuvastatin is highly effective in lowering low‐density lipoprotein‐cholesterol (LDL‐C) and increasing high‐density lipoprotein‐cholesterol (HDL‐C).^21^ Previously, the telmisartan/rosuvastatin single‐pill combination (SPC) has shown non‐inferiority compared to the telmisartan or rosuvastatin monotherapy in lowering brachial BP and LDL‐C levels, respectively.^22^ However, the role of telmisartan/rosuvastatin combination therapy in central BP has not been evaluated yet.

Thus, this study investigated the efficacy and safety of telmisartan/rosuvastatin SPC therapy in patients with HTN and mild dyslipidemia of 10‐year Atherosclerotic Cardiovascular Disease (ASCVD) risk score over 5% compared with telmisartan monotherapy.

## 2 METHODS

### 2.1 Study population

The study population consisted of hypertensive patients aged 50–75 years old, with a calculated ASCVD risk of more than 5%. The participants should also be untreated with dyslipidemia (not under medication or stopped medication for at least 4 weeks prior to randomization). The exclusion criteria were the following: (1) ARB hypersensitivity, (2) secondary HTN, (3) history of myocardial infarction/unstable angina/stroke within 6 months, (4) history of uncompensated congestive heart failure or left ventricular ejection fraction < 40% within 6 months, (5) hemodynamically significant (more than a moderate degree) valvular heart disease, and (6) current serum creatinine > 3 mg/dl, etc.

### 2.2 Study design and procedures

This study was a randomized, multicenter, phase 4, Prospective, Randomized, Open‐label, Blinded End‐point (PROBE) study, which took place from January 11, 2018 to September 2, 2020. All patients were informed thoroughly about the trial and agreed upon written consent. The study protocols were approved by the Institutional Review Board (SNUH 2017‐0412) and were done according to the principles of the Declaration of Helsinki. This study was registered in Clinicaltrials.gov: NCT03267329.

**FIGURE 1:**
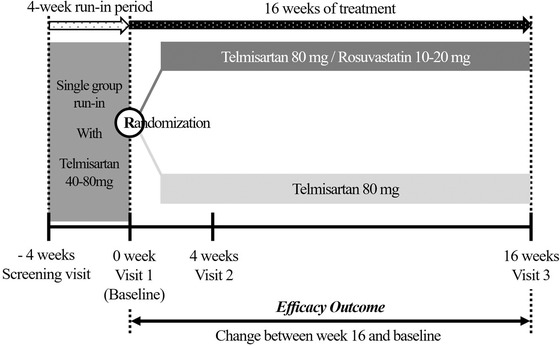
Study design

The patients were randomized after 4 weeks of run‐in period with telmisartan 40 or 80 mg if they met the inclusion criteria. During the run‐in period, the patients who were already taking anti‐hypertensive therapy had their medicine changed to telmisartan 80 mg monotherapy. Others with no experience of anti‐hypertensive therapy were prescribed with telmisartan 40 or 80 mg. After the run‐in period, the patients were randomized via 1:1 stratified randomization by the institute. Depending on the ASCVD risk score during the screening, those with a risk score between 5% and 7.4% received rosuvastatin 10 mg, and those with a risk score above 7.5% received rosuvastatin 20 mg. All medications were taken orally once a day, and the patients were evaluated about the efficacy and safety three times during the 16 weeks of the treatment period (Figure 1).

### 2.3 BP measurement

Electronic BP monitors (Omron HEM‐7080IC, Omron Corporation, Kyoto, Japan) were used to measure brachial BP, and all laboratories used the same product. The BP of both arms was measured by a trained study coordinator at the screening visit after an initial 5 min of seated rest, and the arm with the higher average SBP obtained by three measurements was selected as the reference arm. The average value of the three measurements at screening visit was used as a baseline.

The central hemodynamic parameters were evaluated in the sitting position after 10 min of resting with overnight fasting using a SphygmoCor software version 7.0 (AtCor Medical, Sydney, Australia).^23^ The overnight fasting state was defined as fasting over 8 h. Mean arterial pressure was determined by mathematical integration of the radial pressure waveform and calibrated using the oscillometric value of brachial SBP and DBP. Pulse pressure (PP) amplification was calculated as the ratio of brachial PP: central PP. Brachial‐ankle pulse wave velocity (baPWV) was measured according to the manufacturer's protocol using VP‐1000 (Omron Healthcare CO., Ltd., Kyoto, Japan) after participants were supine for 5–10 min.^24^

### 2.4 Efficacy outcomes

The change in central SBP at 16 weeks from the baseline was evaluated as the primary efficacy outcome (Figure 1). The secondary outcome consisted of the followings: (1) changes of mean brachial SBP (2) changes of AIx (3) changes of baPWV (4) changes of mean brachial PP, and (5) changes of mean brachial DBP at 4 and 16 weeks. The tolerability was evaluated with the occurrence of the adverse reaction, clinical laboratory results, and vital signs.

### 2.5 Sample size and statistical analysis

To estimate the appropriate sample size, the non‐inferiority margin of change of central SBP at 16 weeks was assumed as 4 mmHg with the standard deviation (SD) as 6.8 mmHg. With a significance level of 5% and a power of 80%, the estimated sample size was 46 participants. Assuming the dropout rate of 10%, minimum of 52 participants for each group (a total of 104) was needed for the enrollment.

The intention to treat (ITT) population was defined as participants who had at least one measurement of primary efficacy endpoint among those enrolled after evaluating inclusion/exclusion criteria. Per protocol (PP) set was defined as participants who completed the study without major protocol deviation among the ITT population. The safety set was defined as any of the participants who were treated at least once after randomization. The analysis of baseline characteristics was done at the ITT population. All statistical analyses were done via SAS version 9.4 (SAS Institute, Inc, NC, USA) with a two‐tailed test with a significance level of 5%.

The primary efficacy endpoint of change from baseline in central SBP at 16 weeks was analyzed at the ITT population with paired *t*‐test (or Wilcoxon signed‐rank test) in each treatment group. For between‐group comparison, analysis of covariance was used with baseline central SBP as a covariate. In addition, the between‐group difference of all the adverse events and the adverse drug reaction was analyzed using the Chi‐square test (or Fisher's exact test).

## 3 RESULTS

### 3.1 Patient recruitment and flow

Total 125 patients were screened throughout eight hospitals, and 80 of the patients remained for randomization. In response to the COVID‐19 pandemic from February 2020, there was considerable difficulty in patients’ enrollment. Due to the limitation of study budget, we decided to finish patient enrollment by April 2020. As a result, the number of participants randomized for each group was about 77% of the original plan (A minimum of 52 participants for each group).

**FIGURE 2:**
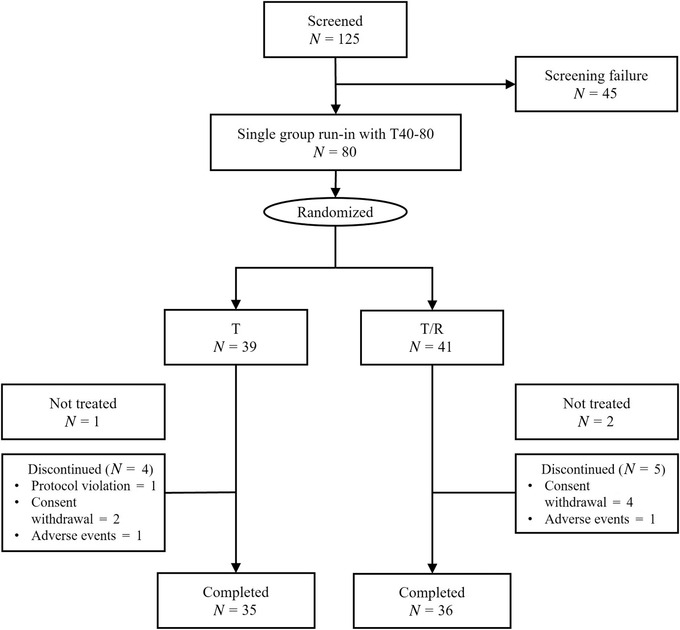
Patient flow. T, telmisartan; T/R, telmisartan/rosuvastatin

Among the screened 125 patients, 45 failed to pass the screening (36 did not qualify inclusion criteria, 8 withdrew consent, 2 other reasons unclarified) and 80 patients went through single‐drug run‐in. Of total 80 single‐drug run‐in population, 71 patients (36 from the telmisartan/rosuvastatin SPC group, 35 from the telmisartan monotherapy group) completed the clinical trial, and 9 (five from the telmisartan/rosuvastatin SPC group, four from the telmisartan monotherapy group) were withdrawn for the following reasons: consent withdrawal (six patients), adverse events (two patients), and protocol violation (one patient) (Figure 2).

### 3.2 Baseline characteristics

Two treatment groups were comparable for all the baseline demographic and clinical characteristics (Table 1). The mean (SD) age was 61.51(9.40), the male proportion of 65%. Mean baseline office SBP/DBP were 135.28(13.76)/82.68(9.52) mmHg. Among the patients, 79.71% were previously treated with anti‐hypertensive medications, most frequently with renin‐angiotensin system inhibitors (71.01%), followed by calcium channel blockers (11.59%). None of the patients in this study population were treated with beta‐blockers. There was no significant difference in previous anti‐hypertensive medication history between two groups. Statins (26.09%), anti‐thrombotic agents (27.54%), oral hypoglycemic agents (17.39%) were the most prevalent drug among other non‐antihypertensive drugs. The dyslipidemia medication, including statin, was discontinued since visit 1 to avoid concomitant usage with clinical trial medications.

**TABLE 1:** Baseline characteristics

Mean ± SD	Telmisartan (*N* = 33)	Telmisartan/rosuvastatin (*N* = 36)	Total (*N* = 69)	*p*‐value
Age (years)	62.15(10.35)	60.92(8.54)	61.51(9.40)	.417^a^
Sex (male %)	22(66.67)	23(63.89)	45(65.22)	.809^b^
Height (cm)	162.00(8.47)	162.53(8.76)	162.28(8.56)	.800^c^
Weight (kg)	69.67(12.72)	67.97(12.35)	68.78(12.47)	.575^c^
Body mass index (BMI, kg/m^2^)	26.47(4.07)	25.60(3.30)	26.01(3.69)	.333^c^
Brachial SBP (mmHg)	134.37(12.50)	135.80(14.22)	135.28(13.76)	.648^a^
Brachial DBP (mmHg)	82.88(8.75)	82.37(9.50)	82.68(9.52)	.862^a^
Pulse rate (BPM)	75.49(10.74)	76.39(11.40)	74.47(11.13)	.736^c^
Smoking (%)	10(30.30)	8(22.22)	18(26.09)	.445^b^
Previous antihypertensive medication (%)	28(84.85)	27(75.00)	55(79.71)	.310^b^
RAS inhibitors	25(75.76)	24(66.67)	49(71.01)	.406^b^
Calcium channel blocker	4(12.12)	4(11.11)	8(11.59)	.896^b^
Diuretics	0(0.00)	1(2.78)	1(1.45)	.335^b^
Other medication (%)	18(54.54)	17(47.22)	35(50.72)	.543^b^
Statins	10(30.30)	8(22.22)	18(26.09)	.445^b^
Antithrombotic agents	11(33.33)	8(22.22)	19(27.54)	.302^b^
Antidiabetic agents	6(18.18)	6(16.67)	12(17.39)	.868^b^

*Abbreviations*: BPM, beats per minute; DBP, diastolic blood pressure; RAS inhibitors, renin‐angiotensin system inhibitors including angiotensin receptor blockers and angiotensin converting enzyme inhibitors; SBP, systolic blood pressure; SD, standard deviation.

^a^ Wilcoxon rank sum test.

^b^ Chi‐square test.

^c^ Two sample *t*‐test.

### 3.3 Changes in brachial and central BP

**FIGURE 3:**
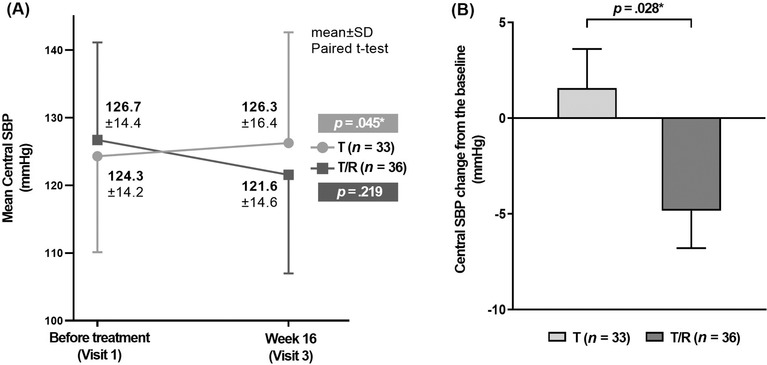
(A) The change of mean central SBP and (B) the least square mean difference of central SBP between the two groups. T, telmisartan; T/R, telmisartan/rosuvastatin; SBP, systolic blood pressure; SD, standard deviation

The primary efficacy endpoint, change of mean central SBP at 16 weeks from baseline, significantly decreased in the telmisartan/rosuvastatin SPC group by −5.17 ± 14.9 mmHg in (*p* = .045), whereas there was no significant change in the telmisartan monotherapy group (+1.94 ± 8.9 mmHg, *p* = .219) (Figure 3A). The least square (LS) mean (standard error) in telmisartan/rosuvastatin SPC group was −4.83(1.96) mmHg in comparison to telmisartan monotherapy group 1.57(2.05) mmHg, resulting in a significant decrease only in the telmisartan/rosuvastatin group. The LS mean difference between the two groups was −6.41 mmHg, indicating a significant reduction in the telmisartan/rosuvastatin group (*p* = .028) (Figure 3B).

The brachial BP, one of the secondary efficacy endpoints, was also evaluated at week 4 and 16. The brachial SBP in telmisartan/rosuvastatin SPC group were significantly reduced in within‐group analysis at week 16 (*p* = .044) but did not show significant difference in between‐group analysis with telmisartan monotherapy group (Table 2). The central DBP and brachial DBP both failed to show significant reduction in either group.

**TABLE 2:** Adjusted changes in central BPs and brachial BPs in the telmisartan/rosuvastatin SPC and telmisartan monotherapy groups

Variables	Visit		Telmisartan (*N* = 33)	Telmisartan/rosuvastatin (*N* = 36)	LS mean difference [95% CI]	*p*‐value
Mean central SBP (mmHg)	Baseline	Mean (SD)	124.33(14.20)	126.72(14.44)		.491^b^
	Week 16	Mean (SD)	126.27(16.36)	121.56(14.56)		
		*p*‐value	.219^d^	.045^d^		
	Change^a^	LS mean (SE)^f^	1.57(2.05)	−4.83(1.96)	−6.41 [−12.08, −0.73]	.028
Mean central DBP (mmHg)	Baseline	Mean (SD)	81.36(10.66)	81.11(12.70)		.929^b^
	Week 16	Mean (SD)	82.48(13.67)	79.92(9.92)		
		*p*‐value	.455^e^	.245^e^		
	Change^a^	LS mean (SE)^f^	1.73(1.89)	−1.26(1.78)	−2.99 [−8.25, 2.26]	.260
Mean brachial SBP (mmHg)	Baseline	Mean (SD)	134.37(12.50)	135.80(14.22)		.648^c^
	Week 16	Mean (SD)	133.75(12.30)	130.69(13.23)		
		*p*‐value	.711^d^	.044^d^		
	Change^a^	LS mean (SE)^g^	−0.98(1.88)	−4.79(1.80)	−3.81 [−9.01, 1.39]	.149
Mean brachial DBP (mmHg)	Baseline	Mean (SD)	82.85(8.75)	82.37(9.50)		.862^c^
	Week 16	Mean (SD)	83.04(11.82)	79.87(10.76)		
		*p*‐value	.886^d^	.064^d^		
	Change^a^	LS mean (SE)^g^	0.21(1.33)	−2.52(1.27)	−2.73 [−6.39, 0.94]	.143

*Abbreviations*: DBP, diastolic blood pressure; LS, least square; PP, pulse pressure; SBP, systolic blood pressure; SD, standard deviation; SE, standard error.

^a^ Change = (Value at week 16) − (value at baseline).

Difference between control and treatment group (^b^two sample *t*‐test, ^c^Wilcoxon rank sum test).

Difference between baseline and post‐baseline in each group (^d^paired *t*‐test, ^e^Wilcoxon signed rank test).

^f^ANCOVA using baseline value as covariate.

^g^Wilcoxon rank sum test.

### 3.4 Changes in baPWV and aortic functional parameters

AIx did not show significant change at week 16 compared with the baseline in both in‐group (*p *= .201 in telmisartan monotherapy group, *p* = .854 in telmisartan/rosuvastatin SPC group) and between‐group analysis (*p* = .314). The baPWV showed decrease in telmisartan/rosuvastatin SPC group although insignificant (from mean 1672.57 m/s to 1591.75 m/s *p* = .134). There was no statistical significance between the two groups in baPWV (between‐group *p* = .078) (Table 3).

**TABLE 3:** Adjusted changes in arterial stiffness assays in the telmisartan/rosuvastatin SPC and telmisartan monotherapy groups

Variables	Visit		Telmisartan (*N* = 33)	Telmisartan/rosuvastatin (*N* = 36)	LS mean difference [95% CI]	*p*‐value
Brachial‐ankle pulse wave velocity (cm/s)	Baseline	Mean (SD)	1542.85(263.70)	1672.57(371.72)		.130^b^
	Week 16	Mean (SD)	1586.12(297.45)	1591.75(272.16)		
	Change^a^	LS mean (SE)^c^	19.70(34.15)	−66.40(33.14)^d^	−86.10 [−182.09, 9.90]	.078
Augmentation index (%)	Baseline	Mean (SD)	35.91(26.15)	33.67(18.32)		.943^b^
	Week 16	Mean (SD)	33.85(26.55)	33.94(19.06)		
	Change^a^	LS mean (SE)^c^	−1.99(1.57)	0.22(1.50)	2.21 [−2.14, 6.56]	.314

*Abbreviations*: DBP, diastolic blood pressure; LS, least square; PP, pulse pressure; SBP, systolic blood pressure; SE, standard error.

^a^ Change = (Value at week 16) − (value at baseline).

^b^ Difference between control and treatment group (Wilcoxon rank sum test).

^c^ Wilcoxon rank sum test.

^d^ *N* = 35.

### 3.5 Changes in biochemical parameters

Serum creatinine, serum potassium, hemoglobin A1C, liver enzyme (aspartate aminotransferase, alanine aminotransferase), high‐density lipoprotein did not show significant difference after 16 weeks compared to the baseline between the two groups. One patient in telmisartan/rosuvastatin SPC group showed abnormal increase in liver enzyme and was reported as ‘hepatic enzyme increase’ adverse effect. The total cholesterol, triglyceride, low‐density lipoprotein of both groups showed significant difference between the two groups at week 16 (*p *< .001, .047, <.001) (Table 4). However, none of the patients showed significant change at the shift table, indicating insignificance of the reduction in the above three values.

**TABLE 4:** Changes of lipid profile at week 16 compared with baseline in the telmisartan/rosuvastatin SPC and telmisartan monotherapy groups

Variables	Visit		Telmisartan (*N* = 38)	Telmisartan/rosuvastatin (*N* = 39)	Total (*N* = 77)
Total cholesterol (mg/dl)	Baseline	Mean (SD)	203.44(35.71)	205.81(40.40)	204.64(37.93)
	Week 16	Mean (SD)	210.68(40.96)	147.23(31.99)	178.52(48.45)
	Change^a^	Mean (SD)	7.09(32.30)	−59.96(36.34)	−26.90(48.03)
		*p*‐value	.196^b^	<.001^b^	<.001^d^
Triglyceride (mg/dl)	Baseline	Mean (SD)	219.87(158.51)	179.18(77.77)	199.26(125.20)
	Week 16	Mean (SD)	193.60(102.82)	155.28(106.41)	174.17(105.68)
	Change^a^	Mean (SD)	6.57(96.78)	−27.33(73.05)	−10.62(86.66)
		*p*‐value	.949^c^	.001^c^	.047^e^
HDL‐cholesterol (mg/dl)	Baseline	Mean (SD)	44.29(9.78)	49.38(11.94)	46.87(11.15)
	Week 16	Mean (SD)	46.22(11.23)	53.83(12.81)	50.03(12.56)
	Change^a^	Mean (SD)	1.36(6.13)	4.25(6.55)	2.81(6.46)
		*p*‐value	.192^b^	<.001^b^	.057^d^
LDL‐cholesterol (mg/dl)	Baseline	Mean (SD)	117.09(44.74)	123.93(35.57)	120.56(40.24)
	Week 16	Mean (SD)	129.10(32.74)	65.32(28.05)	96.30(44.07)
	Change^a^	Mean (SD)	3.82(33.13)	−59.84(34.75)	−28.92(46.53)
		*p*‐value	.506^b^	<.001^b^	<.001^d^

*Abbreviations*: HDL, high density lipoprotein; LDL, low density lipoprotein; LS, least square; SD, standard deviation.

^a^ Change = (Value at week 16) − (value at baseline) .

Difference between baseline and post‐baseline in each group (^b^paired *t*‐test, ^c^Wilcoxon signed rank test).

Difference between control and treatment group (^d^paired *t*‐test, ^e^two sample *t*‐test).

### 3.6 Compliance and tolerability

The overall compliance rate of two groups were 96.14 (SD 4.86)% for the telmisartan group and 97.53 (SD 4.03)% for the telmisartan/rosuvastatin SPC group with no significant difference (*p* = .274).

Of the 77 patients treated with the medication at least once after enrollment, 10 patients experienced 16 adverse events in total. The telmisartan group had five participants with nine events, and the telmisartan/rosuvastatin group had four participants with seven events. The incidence rate between the two groups did not differ significantly (*p* = 1.000). A major adverse event was found once at one participant in the telmisartan group: a renal cyst (not an adverse drug reaction). The adverse event that resulted in discontinuation of the treatment was found once each for both telmisartan and telmisartan/rosuvastatin group (*p* = 1.000). The most common adverse event was asthenia, which was found in a total of two participants, one from each group. The adverse event that resulted in the treatment's discontinuation was a headache in the telmisartan group, and hepatic enzyme increase in the telmisartan/rosuvastatin group. The headache was considered as an adverse drug reaction related to the clinical trial medication.

## 4 DISCUSSION

In this study, we found that the telmisartan/rosuvastatin SPC group had more significantly reduced mean central SBP than the telmisartan monotherapy group in patients with HTN and mild dyslipidemia. The brachial SBP were also reduced in telmisartan/rosuvastain SPC group but without significance compared to telmisartan monotherapy group. The between‐group difference of AIx and baPWV were statistically insignificant despite the baPWV being reduced only in telmisartan/rosuvastatin SPC group.

Although statin's effect on lowering BP has been identified previously, its effect on lowering central BP is still controversial. In a small‐scale randomized controlled trial, the statin usage reduced central BP and aortic stiffness.^25^ However, in the CAFE‐LLA study, statin did not lower central BP.^26^ Most recently, in the study from the CARTaGENE cohort, the statin use was significantly associated only in patients who were targets of primary prevention.^27^ This result is in line with our study as all the participants in this study were at least over 5% of the ASVCD risk factor, which makes them the target of primary prevention.

The different result between the two groups might partly be explained by the withdrawal of statin in 30% of participants who were previously prescribed with statin in the telmisartan monotherapy group. Previous studies have shown the withdrawal of statin to be associated with increased risk of stroke or acute coronary syndrome.^28^, ^29^ However, those studies were focused on the acute phase of vascular stress with inclusion criteria of patients who had stroke or coronary artery disease in the previous 24 h, respectively.^28^, ^29^ On the other hand, study by McGowan showed no clinically important increase of acute coronary syndrome in the stable cardiac patients who went through short‐term statin withdrawal.^30^ As our study population excluded any participants who had acute coronary syndrome or stroke within 6 months, the withdrawal of statin would not have affected as much as the previous studies by Blanco and colleagues or Heeschen and colleagues but rather be similar to study by McGowan. Also, approximately 75% of study population were never treated with statin before, thus the difference cannot solely be explained by the effect of withdrawal of statin.

Although insignificant in between‐group analysis, the baPWV of the telmisartan/rosuvastatin SPC group showed greater reduction at week 16 from the baseline compared to the telmisartan monotherapy group. Such reducing tendency of baPWV might have contributed to the decrease in central BP. There are not many studies that reveal the association between reduced PWV and reduced central BP. Study by Hirata et al. (2005) and Raff et al. (2015) are one of the few studies showing decrease in PWV and central BP after use of ramipril and olmesartan, respectively.^31^, ^32^

PWV is the most known measure of arterial stiffness, along with AIx.^33^ Arterial stiffness is caused by hemodynamic forces, extrinsic factors including hormones, and inflammations.^34^ The meta‐analysis of 52 studies done by McGaughey and colleagues have shown that ACEIs and ARBs can significantly reduce AIx.^35^ Furthermore, meta‐analysis by Yen and colleagues suggested that ARBs can also reduce PWV.^36^ Not only ACEIs and ARBs but also meta‐analyses on statin have significantly reduced the aortic stiffness, defined by PWV and AIx.^37^, ^38^, ^39^ Such effect of the drugs is explained by renin‐angiotensin‐axis system mechanism of ARBs and antioxidant and anti‐inflammatory activity of statin.^36^, ^40^

As the telmisartan monotherapy group showed no reduction in PWV at all, the reducing tendency shown at telmisartan/rosuvastatin SPC group might have been due to the addition of statin. The effect of statin on PWV is expected to be dose dependent.^41^ Thus, the statistical significance of statin on PWV in this trial might have been attenuated due to the mixture of rosuvastatin dosage 10  and 20 mg. In this study, the reduction of PWV showed marginal significance with AIx having no statistically significant difference between the two groups. Such result could be due to different effect of treatment duration on PWV and AIx. The meta‐analysis by Sahebkar and colleagues found positive association between statin treatment duration and AIx.^39^ On the other hand, the meta‐analysis by D'elia and colleagues and Upala and colleagues found reduction of PWV despite the difference of statin treatment duration.^37^, ^38^ Thus, the discordance on the change of baPWV and AIx may be due to baPWV being independent from treatment duration but AIx being more dependent on the duration. Also, although study by Van Doornum and colleagues showed reduction of AIx in rheumatoid arthritis patients after 12 weeks of atorvastatin, the potency of statin is thought to differ between the type of statin.^39^, ^42^ Thus, appropriate duration needed for the reduction of AIx might differ in rosuvastatin. Furthermore, AIx is known to be affected by age, showing different response according to age, reaching plateau at age of 60 years.^43^ This difference makes AIx more sensitive marker in younger age group and less feasible to elders. Study by Hayashi and colleagues even showed that AIx might not be an appropriate marker to identify coronary artery disease in elderly.^44^ On the other hand, PWV increases along with age even after 50 years.^45^ And the aging influences on baPWV more prominently in female.^46^ As the mean age of our study population was 61.5 years, the AIx might not have been a suitable marker leaving baPWV as a more feasible one. This is one of the limitations of our study, and further study comprising younger age group would be necessary to clear this point.

The cause why BP lowering at week 16 compared to baseline in telmisartan monotherapy group was insignificant, might come from the 4‐week single group run‐in period with telmisartan 40–80 mg. As our study protocol comprised of 4‐week run‐in period with telmisartan 40 or 80 mg, most participants in telmisartan monotherapy group continued their run‐in period treatment without change. Therefore, it is not surprising that there was no significant change in BP between the baselines to week 16. Actually, among the 33 participants who were in telmisartan monotherapy group, only five participants were prescribed with telmisartan 40 mg in the run‐in period and up‐titrated to telmisartan 80 mg during the 16 weeks of treatment. Other 28 participants were on telmisartan 80 mg since run‐in period. Among the five participants who had telmisartan dosage changed, three participants showed further BP reduction whereas two did not show any reduction of BP (data not shown). This proportion of patients who had telmisartan dosage up‐titrated in the treatment period might be too small to affect significance of within‐group reduction.

Pulse pressure amplification (PPA), defined by ratio of brachial PP to central PP, is another factor known to be inversely associated with cardiovascular risk and mortality.^7^, ^47^ Decrease in central SBP caused by statin as shown in this study would eventually give rise to PPA ultimately resulting in reduced cardiovascular risk and mortality.

The main limitation of this study was a premature closure of enrolment, restricting the study as a pilot study. Although the investigators tried every effort to complete enrollment, the COVID‐19 crisis, which has continued in the current situation, significantly disabled patient's visit in the tertiary hospitals, which were recruited to COVID‐19 patient's care. Therefore, after deep consideration, the investigators and the sponsor decided to finish the patient enrollment in April 2020. This premature termination of patient enrollment unfulfilled the statistically calculated population, which is the major limitation of this study.

Because of the small study population, the result was interpreted with careful consideration of the possible weak points derived by the small study design. As a small study, the probability of false positive (type 1 error) is increased and over‐estimation of the magnitude of association can be found more easily.^48^ Not only type 1 error, but also previous evaluation on randomized controlled trials also showed a need for caution to the occurrence of type 2 error in underpowered randomized controlled trials.^49^ Consequently, the result of our study cannot be confirmative and necessitates the need for larger confirmatory study.

The marginal significance of PWV mostly came from the smaller sample size than originally planned. Besides underpowered study, the marginal significance of PWV may be due to the other factors that influence PWV. The PWV is known to be influenced by PP, age, sex, BP, body mass index, triglyceride, blood glucose, salt intake, electrocardiogram voltage, urine albumin, and genetic factors.^50^, ^51^, ^52^ In this study, the two groups did not differ between the PP, age, and sex. However, other factors including salt intake, urine albumin, genetic factors were not considered. Thus, such factors might have attenuated the PWV difference in this study. This is one of the limitations of our study, and further study comprising younger age group would be necessary to clear this point.

On the contrary, there is also a chance that the effect has been exaggerated due to higher baseline baPWV in telmisartan/rosuvastatin SPC group. Although the participants were randomly assigned to each group, there was numerical difference in baseline PWV yet without statistical significance (*p* = .130). We did not exclude the possibility that the difference might be significant if the original enrolment was completed (type 2 error). However, telmisartan the monotherapy group showed numerically increased PWV by 2.9%, whereas the telmisartan/rosuvastatin SPC group showed decreased PWV by 4.8%. Therefore, even though the extent of change might be influenced by the baseline values, we think that the difference between two groups remains significant.

## 5 CONCLUSIONS

In this study, telmisartan/rosuvastatin combination therapy showed a reduction in both central and brachial SBP when the telmisartan monotherapy group failed to show significant reduction in patients with HTN and mild dyslipidemia. The results of this study showed another benefit of statin therapy in hypertensive patients combined with dyslipidemia.

## CONFLICT OF INTEREST

The authors have indicated that they have no conflicts of interest regarding the content of this article.

## AUTHOR CONTRIBUTIONS

This study was coordinated by Hae‐Young Lee as the principal investigator. Ki‐Chul Sung, Sang‐Hyun Ihm, Chang‐Hwan Yoon, Seung Woo Park, Sung‐Ha Park, Jang‐Young Kim, and Sung‐Uk Kwon contributed equally to data collection and validation. Jung Min Choi contributed to writing and original draft preparation.
